# Acute-on-chronic liver failure alters meropenem pharmacokinetics in critically ill patients with continuous hemodialysis: an observational study

**DOI:** 10.1186/s13613-020-00666-8

**Published:** 2020-04-22

**Authors:** Jörn Grensemann, David Busse, Christina König, Kevin Roedl, Walter Jäger, Dominik Jarczak, Stefanie Iwersen-Bergmann, Carolin Manthey, Stefan Kluge, Charlotte Kloft, Valentin Fuhrmann

**Affiliations:** 1 Department of Intensive Care Medicine, University Medical Center Hamburg-Eppendorf, Martinistraße 52, 20246 Hamburg, Germany; 2 Department of Clinical Pharmacy and Biochemistry, Institute of Pharmacy, Freie Universitaet Berlin, Kelchstraße 31, 12169 Berlin, Germany; 3 Graduate Research Training Program PharMetrX, Berlin, Germany; 4 Hospital Pharmacy, University Medical Center Hamburg-Eppendorf, Martinistraße 52, 20246 Hamburg, Germany; 5 Department of Pharmaceutical Chemistry, University of Vienna, Althanstraße 14, 1090 Vienna, Austria; 6 Department of Legal Medicine, University Medical Center Hamburg-Eppendorf, Butenfeld 34, 22529 Hamburg, Germany; 7 First Department of Internal Medicine and Gastroenterology, University Medical Center Hamburg-Eppendorf, Martinistraße 52, 20246 Hamburg, Germany; 8 Department of Medicine B, Münster University Hospital, Albert-Schweitzer-Campus 1, 48149 Münster, Germany

**Keywords:** Antibiotics, Target attainment, Intensive care, Volume of distribution, Monte Carlo simulation, Population pharmacokinetics, Probability of target attainment

## Abstract

**Background:**

Infection and sepsis are a main cause of acute-on-chronic liver failure (ACLF). Adequate dosing of antimicrobial therapy is of central importance to improve outcome. Liver failure may alter antibiotic drug concentrations via changes of drug distribution and elimination. We studied the pharmacokinetics of meropenem in critically ill patients with ACLF during continuous veno-venous hemodialysis (CVVHD) and compared it to critically ill patients without concomitant liver failure (NLF).

**Methods:**

In this prospective cohort study, patients received meropenem 1 g tid short-term infusion (SI). Meropenem serum samples were analyzed by high-performance liquid chromatography. A population pharmacokinetic analysis was performed followed by Monte Carlo simulations of (A) meropenem 1 g tid SI, (B) 2 g loading plus 1 g prolonged infusion tid (C) 2 g tid SI, and (D) 2 g loading and continuous infusion of 3 g/day on days 1 and 7. Probability of target attainment (PTA) was assessed for 4× the epidemiological cut-off values for *Enterobacterales* (4 × 0.25 mg/L) and *Pseudomonas* spp. (4 × 2 mg/L).

**Results:**

Nineteen patients were included in this study. Of these, 8 patients suffered from ACLF. A two-compartment model with linear clearance from the central compartment described meropenem pharmacokinetics. The peripheral volume of distribution (*V*_2_) was significantly higher in ACLF compared to NLF (38.6L versus 19.7L, *p* = .05). PTA for *Enterobacterales* was achieved in 100% for all dosing regimens. PTA for *Pseudomonas* spp. in ACLF on day 1/7 was: A: 18%/80%, B: 94%/88%, C: 85%/98% D: 100%/100% and NLF: A: 48%/65%, B: 91%/83%, C: 91%/93%, D: 100%/100%.

**Conclusion:**

ALCF patients receiving CVVHD had a higher *V*_2_ and may require a higher loading dose of meropenem. For *Pseudomonas*, high doses or continuous infusion are required to reach PTA in ACLF patients.

## Background

Sepsis and septic shock are frequent complications in patients with acute-on-chronic liver failure (ACLF) and associated with a high mortality [1–7]. Early empiric broad-spectrum antibiotic therapy is indicated in these critically ill patients [8]. Meropenem is often used for this purpose due to its broad spectrum and still favorable resistance profile. Acute kidney injury is the most frequent type of organ failure in ACLF [3] requiring renal replacement therapy in this patient population [9]. Attaining sufficient antibiotic concentrations is important for the therapy of sepsis. Due to an increased volume of distribution (*V*) as a result of capillary leak syndrome and a decreased elimination due to organ dysfunction, antibiotic pharmacokinetics (PK) is highly variable in critical illness. Therefore, dosing recommendations for meropenem during renal replacement therapies range from 0.5 to 3 g per day [10]. Concerning hepatic insufficiency, only PK and pharmacodynamic data from patients with stable alcoholic cirrhosis are available [11], but to date, there are no available data in patients suffering from ACLF with multiorgan failure.

Therefore, we studied the impact of ACLF on PK of meropenem in critically ill patients requiring continuous veno-venous hemodialysis (CVVHD) in comparison to critically ill patients receiving CVVHD without ACLF.

## Methods

### Ethics

The study was approved by the Ethics Committee of the Hamburg Chamber of Physicians, Germany (Reference: PV5415). Consent was obtained from the patients’ closest relatives or legal surrogates.

### Study design

Patients eligible for this open-label observational prospective cohort study were receiving meropenem for clinical indication and required CVVHD. Patients < 18 years or with an extracorporeal circuit other than the CVVHD were excluded. Patients were grouped according to liver function as follows: patients with ACLF and patients without ACLF (“no liver failure”, NLF).

### Liver cirrhosis and ACLF

ACLF was defined according to the definition of the Chronic Liver Failure (CLIF) Consortium [3]. Presence of liver cirrhosis was diagnosed based on a combination of characteristic clinical (e.g., ascites, caput medusae, spider angiomata, etc.), laboratory and radiological findings (typical morphological changes of the liver, signs of portal hypertension, etc., in ultrasonography or computed tomography scanning), or via histology, if available [12].

### Medication

All patients received meropenem 1 g quid 8 h (Dr. Friedrich Eberth Arzneimittel GmbH, Germany). Meropenem was diluted in 50 mL isotonic saline solution and given over 30 min by syringe pump via a central venous line (short-term infusion).

### Sampling and storage

We obtained prefilter blood samples at the following time points: T0 as the baseline before the first monitored infusion, 1 h (T1), 2 h (T2), 4 h (T4), 6 h (T6) and 8 h after the start of infusion (T8). T8 was obtained before the next infusion of meropenem as a minimum concentration. Furthermore, we obtained values after 24 h (before and 30 min after end of infusion, T24 and T25) and after 48 h (T48 and T49). All samples were centrifuged immediately, and the supernatant stored at − 20 °C until assayed.

### Assay

An aliquot of a human serum sample (250 µL) was mixed with 50 µL of the internal standard ertapenem (0.2 mg/mL). 500 µL acetonitrile was added to precipitate serum proteins. After centrifugation at 14500 rpm for 5 min at 20 °C, 100 µL of the supernatant was diluted to a total of 1000 µL with water in a glass microvial. The injection volume was 50 µL.

A validated high-performance liquid chromatography with diode array detection (HPLC–DAD) method was used for the analysis of meropenem serum samples. Chromatography was performed on a Thermo Scientific Accela Liquid chromatography system consisting of an autosampler, quaternary pump and a photo diode array detector with a thermostated column compartment (Thermo Fisher Scientific, Waltham, MA, USA). The chromatographic separation of meropenem and internal standard was carried out on a reversed phase Varian Polaris C18-A (250 × 4.0 mm) with particle size of 5 µm and a C18-pre-column (SecurityGuard^™^ Phenomenex^®^, Aschaffenburg, Germany) using a flow rate of 1.0 mL/min at 25 °C. The mobile phases consisted of water with 0.2% ortho-phosphoric acid added (A) and acetonitrile (B). The following gradient program was used (proportion of solution (B): 5% at 0.0 min, 20% at 6.0 min, 70% at 11.0 min and 5% at 14.0 min. Between injections, the sampling needle was flushed with 400 µL and washed with 200 µL methanol. The wavelength detection was set at 260 nm and 310 nm. Chromeleon™ 7 Chromatography Data Systems Software (Thermo Fisher Scientific, Waltham, MA, USA) was used for the control of the instruments and data acquisition. The assay was routinely calibrated using six standards of spiked blank human serum (1, 10, 20, 50, 100, 150 mg/L) and there were also two independently prepared quality control samples (16 and 64 mg/L) included in each analytical series. Validation parameters including accuracy, interferences, linearity of calibration, matrix effects and in-process stability complied with international standards. Regular external quality control is performed by periodical proficiency testing. The limit of quantification was 1 mg/L.

### Continuous veno-venous hemodialysis

CVVHD was performed with Multifiltrate^®^ dialysis machines using an Ultraflux^®^ AV1000S hollow-fiber hemofilter (Fresenius Medical Care, Bad Homburg, Germany) with a membrane surface area of 1.8 m^2^. Dialyzers and lines were steam sterilized. A regional citrate-calcium anticoagulation was used. No filter change occurred during the study period. The targeted dialysate dose was 30 ml/kg/h of actual body weight.

### Patient characteristics

Additional data were obtained from the patients’ electronic records (Integrated Care Manager ICM, version 9.1, Drägerwerk, Lübeck, Germany, and Soarian Clinicals 4.01 SP08, Cerner Health Services, Idstein, Germany).

The Acute Physiology and Chronic Health Evaluation II (APACHE II) score [13] and the Sequential Organ Failure Assessment (SOFA) score [14] were recorded on the first day of examination as measures of disease severity. ACLF patients were further characterized by the Model of End-Stage Liver Disease (MELD) score, the Chronic Liver Failure Consortium (CLIF)-SOFA score, and the CLIF-lactate score [12].

### Statistics

Microsoft Excel 2016 (Microsoft Corp., Redmond, WA, USA) was used for data management. The SPSS statistical software package (version 25, IBM Inc., Armonk, NY, USA) was used for descriptive statistical analysis. The pharmacokinetic analysis and Monte Carlo analysis were done with the non-linear mixed-effects modeling software NONMEM, version 7.4 (Icon Development Solutions, Ellicott City, MD, USA). Data are given as median and quartiles.

### Pharmacokinetic analysis

The population PK analysis comprised the development of a structural and statistical base model followed by a categorical evaluation of PK parameter differences between the ACLF and NLF patient population. One and two-compartment PK models with serum data attributed to the central compartment were evaluated. Elimination from the central compartment and intercompartmental distribution were assessed using linear processes via ordinary differential equations. Interindividual variability as described by the coefficient of variation of the exponential model was implemented on clearance, intercompartmental clearance, and peripheral volume of distribution (*V*_2_). Interindividual variability in PK parameters, and different residual unexplained variability models to quantify the difference between the model predictions and observations were investigated. The informativeness of interindividual variability and residual unexplained variability parameters was assessed based on shrinkage [15]. The model quality was evaluated by visual inspection of the observed and predicted meropenem concentrations, residuals and individual predicted PK parameter scatter plots as well as visual predictive checks (*n* = 1000 simulations) [16]. Statistical comparisons between nested models with additional covariates were made using the likelihood-ratio test [17]. Because of limited data for the first declining phase of the meropenem concentration–time profile and large parameter imprecision and parameter correlation between intercompartment clearance and central volume of distribution (*V*_1_), *V*_1_ was fixed at 8.31 L as plausible value previously described for critically ill patients [18].

### Probability of target attainment (PTA)

Based on the PK parameters of the final population PK model, Monte Carlo simulations (*n* = 1000) were performed to determine the PTA for different meropenem dosing regimens in ACLF and NLF patients. PTA was defined as time above 4× the European Committee on Antimicrobial Susceptibility Testing (EUCAST) epidemiological cut-off values (ECOFF) as typical minimum inhibitory concentration (MIC) values [19, 20] of the free fraction of meropenem (*f*T > _4×ECOFF_). PTA for achieving a PK/pharmacodynamic target of 95% of *f*T > _4×ECOFF_ was calculated on the first day of the therapy and of 100% *f*T > _4×ECOFF_ at steady-state on day 7. A dosing regimen was considered adequate if the PTA was ≥ 90%.

PTA was calculated for the following ECOFFs: 0.25 mg/L for *Enterobacterales* and 2.0 mg/L for *Pseudomonas aeruginosa* or *Acinetobacter baumannii* and the following dosing regimens: meropenem 1 g short-term infusion (over 30 min) quid 8 h, 1 g quid 8 h as prolonged infusion over 4 h after a short-term infusion loading dose of 2 g and without loading dose, 2 g short-term infusion quid 8 h, and continuous infusion of meropenem 3 g/day after a short-term infusion loading dose of 2 g.

## Results

A total of 19 critically ill patients were included in this study with eight patients suffering from ACLF and renal failure and 11 patients only from renal failure. ACLF patients had a mean MELD score of 34 (27–37), a CLIF-SOFA of 15 (14–15) and a CLIF-lactate score of 59 (57–64). An overview of the patients’ characteristics is given in Table 1. Two ACLF patients were admitted for variceal hemorrhage, two for pneumonia, two for spontaneous bacterial peritonitis, one for urosepsis, and one for hepatorenal syndrome. Gram-positive bacteria were identified in four patients in microbiological sampling, *Escherichia coli* and *Candida* spp. in one case each, and no pathogens were found in two patients. In the NLF group, five patients were treated for hospital acquired pneumonia, four patients for peritonitis and one patient each for cholecystitis and for complicated soft tissue infection. Microbiological sampling detected two cases of *Pseudomonas aeruginosa*, *Enterobacter cloacae*, *Klebsiella pneumoniae*, *Serratia marcescens*, and yeasts in one case each, Gram-positive bacteria in four patients, and was without results in three patients. Seven patients (88%) suffering from ACLF and eight (73%) patients in the NLF group died during the intensive care stay (*p* = 0.60). Ascites was present in 75% of ACLF patients.

**Table 1:** Patients’ characteristics

Parameter	ACLF	NLF	*p*
Number of patients	*n* = 8	*n* = 11	
Age [years]	59 (46–68)	61 (55–75)	0.31
Gender	Males: 6	Males: 8	1.0
	Females: 2	Females: 3	
Weight [kg]	78 (60–81)	77 (55–86)	0.97
Height [cm]	175 (166–180)	175 (170–184)	0.49
APACHE II	30 (25–40)	32 (20–38)	0.97
SOFA	16 (15–19)	14 (11–19)	0.44
Blood flow rate [mL*h^−1^]	100 (100–200)	100 (100–200)	0.84
Dialysate rate [mL*h^−1^]	2000 (2000–2750)	2000 (2000–2000)	1.0
Ultrafiltrate [mL*h^−1^]	25 (0–138)	150 (50–200)	0.17
Dialysate dose [ml*kg^−1^*h^−1^]	31 (23–37)	26 (23–40)	0.90
Patients receiving norepinephrine ≥ 0.01 µg*kg^−1^*min^−1^	88%	73%	0.60
PT [%]	50 (29–61)	87 (74–103)	0.001
Bilirubin [mg/dL]	7.4 (4.3–18.7)	2.1 (0.4–8.3)	0.05
Antithrombin [%]	48 (22–54)	71 (56–90)	0.004

*ACLF* acute-on-chronic liver failure due to liver cirrhosis, *NLF* patients without liver failure, *APACHE II* Acute Physiology and Chronic Health Evaluation, *SOFA* Sequential Organ Failure Assessment Score, *PT* prothrombin time, *ns* not statistically significant, data are given as median and quartiles

### Meropenem serum concentrations and pharmacokinetic model

In total, 19 meropenem measurements were available per sampling time point for the first study dosing interval on day 1 and day 2 with values missing for T4 to T8 for 2 patients and 17 measurements per sampling time point available on day 3 (*n*_total_ = 180). The meropenem clearance of the CVVHD was 4.99 (4.32–5.75) L/h.

Meropenem PK in patients with and without ACLF was described by a two-compartment PK model with linear clearance from the central compartment. Interindividual variability for clearance was coefficient of variation = 28% and on *V*_2_ = 66%. Separate estimation of *V*_2_ yielded a *V*_2_ of 18.5 L for NLF patients and 35.5 L for ACLF patients (*p* = 0.05, likelihood-ratio testing), which largely explained interindividual variability in *V*_2_ (61.9% relative reduction). The remaining interindividual variability in *V*_2_ was estimated imprecisely and suffered from a large shrinkage (50.8%), indicating non-informativeness of this parameter by the data. Due to the model stability, it was excluded in the final model. No difference in clearance between the two patient populations was evident (ACLF: 5.20 L/h, NLF: 5.11 L/h; *p *= 0.90). The half-lives of ACLF compared to NLF patients were *t*_½α_: 0.43 vs. 0.40 h (*p* = 0.60) and *t*_½β_: 9.0 vs. 5.0 h (*p* = 0.002). The final model parameter values are depicted in Table 2.

**Table 2:** Parameter estimates for meropenem from the final covariate two-compartment population pharmacokinetic model

Parameter	ACLF	NLF	p
Structural pharmacokinetic parameters for meropenem (RSE, %)			
CL [L/h]	5.06 (6.70)		ns
*V*_1_ [L]	8.31^a^		n/a
*V*_2_ [L]	35.5 (33.3)	18.5 (33.2)	0.05
*Q* [L/h]	7.23 (9.80)		n/a
Interindividual variability parameters for meropenem (RSE, %)			
CL, CV [%]	29.8 (16.8)		n/a
*Q*, CV [%]	27.6 (28.8)		n/a
Residual variability parameter σ proportional, CV %	22.0 (8.40)		n/a

RSE is presented on the approximated standard deviation scale

*ACLF* acute-on-chronic liver failure, *NLF* patients without liver failure, *CL* clearance, *V*_*1/2*_ central/peripheral volume of distribution, *Q* intercompartmental clearance, *CV* coefficient of variation, *ns* not statistically significant, *n/a* not applicable, *RSE* relative standard error

^a^Value fixed (see “Methods” section)

### Target attainment

**Fig. 1:**
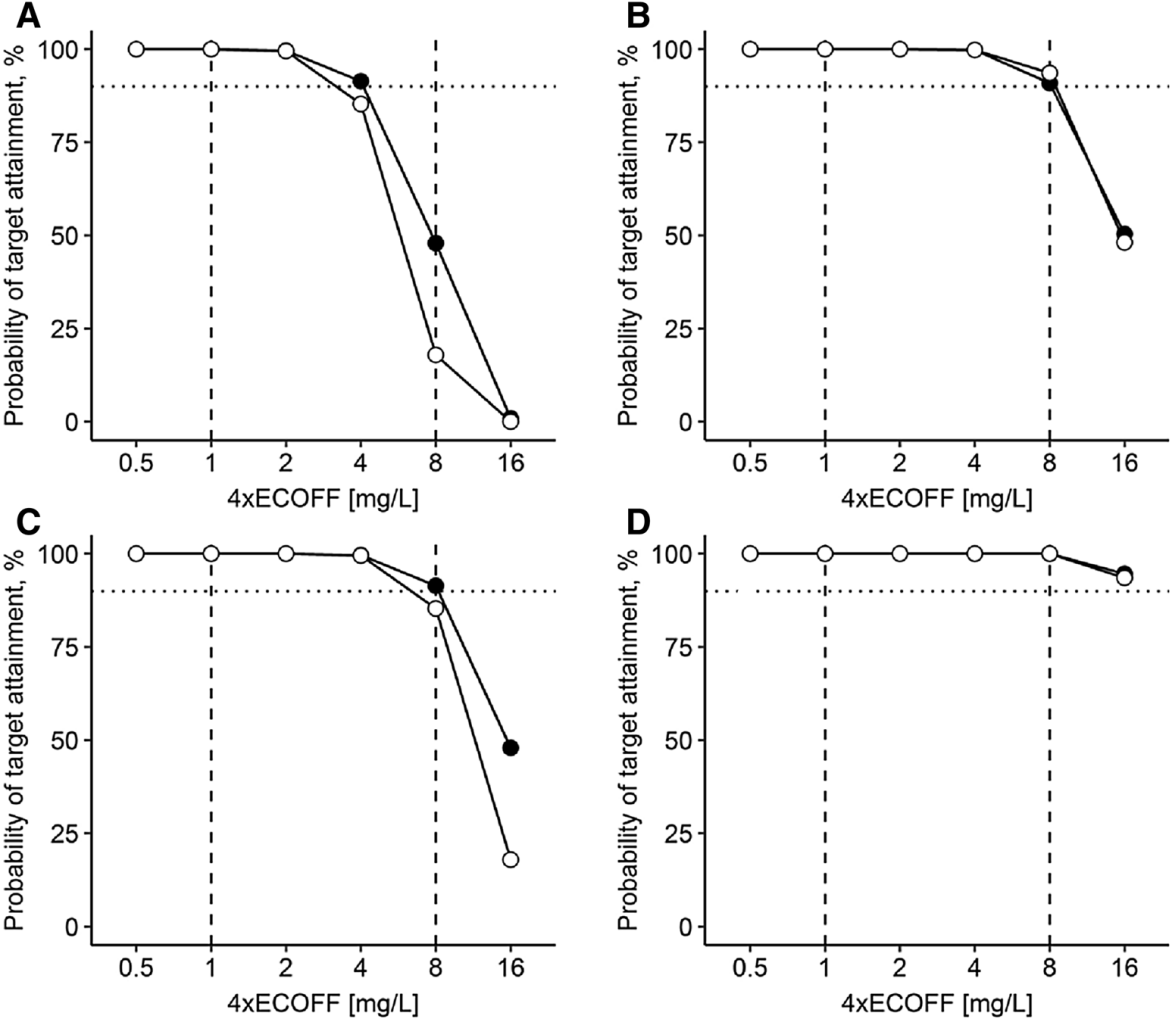
Probability of target attainment on day 1. **a** 1 g quid 8 h (30 min); **b** 2 g loading dose (30 min) followed by 1 g prolonged infusion (over 4 h) quid 8 h; **c** 2 g quid 8 h (30 min); **d** 2 g loading dose (30 min) followed by continuous infusion of 3 g/day. Empty dots: patients with acute-on-chronic liver failure, closed dots: patients without liver failure, dotted horizontal line depicts probability of target attainment ≥ 90%, dashed vertical lines depict typical target concentrations as 4x European Committee on Antimicrobial Susceptibility Testing epidemiological cut-off values (ECOFF) for *Enterobacterales* (4× ECOFF 0.25 mg/L = 1 mg/L) and *Pseudomonas* spp. (4× ECOFF 2.0 mg/L = 8 mg/L). *MIC* minimum inhibitory concentration

**Fig. 2:**
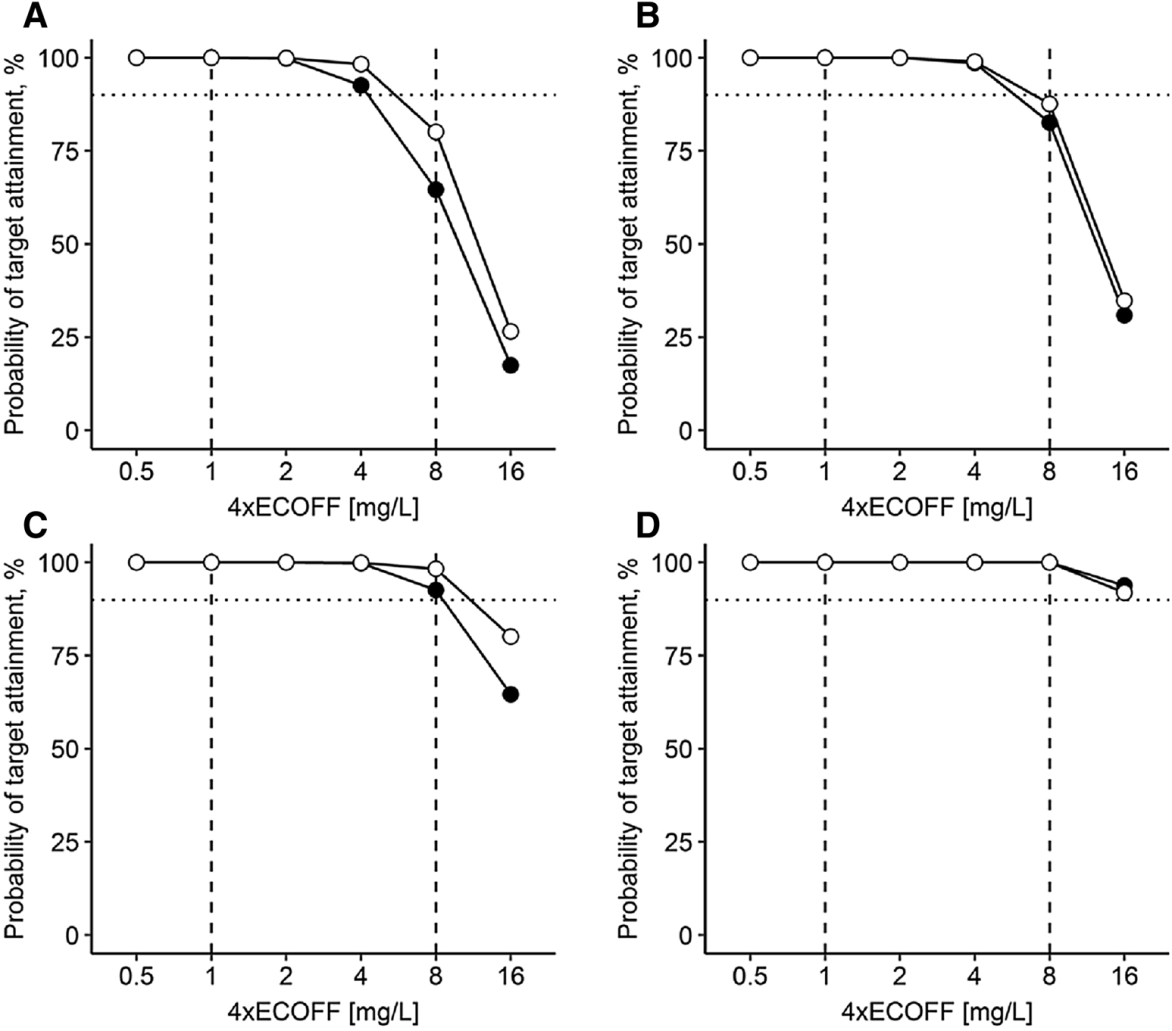
Probability of target attainment at steady-state (day 7). **a** 1 g quid 8 h (30 min); **b** 2 g loading dose (30 min) followed by 1 g prolonged infusion (over 4 h) quid 8 h; **c** 2 g quid 8 h (30 min); **d** 2 g loading dose (30 min) followed by continuous infusion of 3 g/day. Empty dots: patients with acute-on-chronic liver failure, closed dots: patients without liver failure, dotted horizontal line depicts probability of target attainment ≥ 90%, dashed vertical lines depict typical target concentrations as 4x European Committee on Antimicrobial Susceptibility Testing epidemiological cut-off values (ECOFF) for *Enterobacterales* (4× ECOFF 0.25 mg/L = 1 mg/L) and *Pseudomonas* spp. (4× ECOFF 2.0 mg/L = 8 mg/L). MIC: minimal inhibitory concentration

PTA for the lower ECOFF of 0.25 mg/L (*Enterobacterales*) was 100% for all dosing regimens on day 1 (Fig. 1) and on day 7 (Fig. 2) in both groups. For the higher ECOFF of 2.0 mg/L (*P. aeruginosa, A. baumannii*), meropenem 1 g short-term infusion quid 8 h yielded a PTA of 18% in the ACLF group and 48% in the NLF group on day 1 and 80% for ACLF and 65% for NLF patients on day 7. With prolonged infusion of 1 g quid 8 h, PTA was increased to 30% and 67% on day 1 and 88% and 81% on day 7 for ACLF and NLF patients, respectively. For a prolonged infusion after a short-term infusion loading dose of 2 g, PTA was higher with 94% and 91% on day 1 and 88% and 83% on day 7. The dosing regimen of 2 g short-term infusion quid 8 h yielded a PTA of 85% and 91% on day 1 and 98% and 93% on day 7. The continuous infusion of 3 g quid 8 h after a short-term infusion loading dose of 2 g reached a PTA of 100% for both groups on days 1 and 7.

Therefore, adequate PTA was achieved for all dosing regimens up to an ECOFF of 0.25 mg/L. However, for the higher ECOFF of 2 mg/L, only a 2 g short-term infusion followed by a continuous infusion of 3 g/day reached adequate PTA on day 1 and 7 in both groups. A dosing regimen of 2 g quid 8 h was sufficient on day 1 only in the NLF group and in both groups at steady-state.

## Discussion

In this study, we assessed the impact of ACLF on PK and PTA of meropenem in critically ill patients undergoing CVVHD. For the ECOFF values typical for *Enterobacterales*, all simulated dosing regimens yielded an adequate PTA. However, for non-fermenting bacteria with higher ECOFF values like *Pseudomonas* spp. or *Acinetobacter* spp., the standard dosing regimen of 1 g quid 8 h failed to achieve adequate PTA at start and steady-state in ACLF. In contrast, dosing regimens of 2 g short-term infusion quid 8 h or the continuous dosing regimen of 3 g/day showed adequate PTA at steady-state. Additionally, the larger *V*_2_ in ACLF patients required higher loading doses to achieve similar PTA compared to NLF patients during day 1.

Meropenem is often used as empiric broad-spectrum antimicrobial therapy in patients with ACLF suffering from sepsis and septic shock. These critically ill patients often suffer from multiple organ failure and receive renal replacement therapy. The Food and Drug Administration (FDA) label states that insufficient data are available for an informed adjustment of the dosing regimen of meropenem on renal replacement therapy, but several small studies have evaluated the PK on different types of renal replacement therapy by now [21–24]. According to both FDA and European Medicines Agency (EMA) labeling, no adjustment of the dosing regimen is required in patients with hepatic impairment. However, these recommendations are based on data obtained in non-critically ill patients with single organ failure and do not take into account the complex changes of distribution and elimination kinetics in ACLF [11]. Furthermore, antibiotic therapy must “hit hard” especially in the initial phase to prevent an increase in sepsis-related mortality, foremost in ACLF patients [25] and insufficient antibiotic concentrations have been linked to an increase in mortality, particularly in critically ill patients [26].

In a recent meta-analysis, a decrease in mortality has been shown for prolonged or continuous meropenem infusions that achieve concentrations for a longer time above the MIC as compared to bolus applications [27] and has been recently recommended in critically ill patients [28]. Therapeutic drug monitoring has been proposed to detect insufficient antibiotic concentrations and to allow for adjustments in the dosing regimen, but therapeutic drug monitoring is not ubiquitously available and often does not allow for immediate adjustments after the initiation of antibiotic therapy. Therefore, PK models predicting drug distribution and elimination kinetics are useful in the critical care setting until therapeutic drug monitoring results allowing for targeted adjustments are available.

Currently, there are no PK data available in patients with ACLF. It has been shown for other beta-lactam antibiotics that V increases in patients with liver cirrhosis and ascites [29, 30] and that meropenem is readily redistributed into the peritoneal fluid in septic shock [31]. Hence, our finding of a nearly twice as high V_2_ for ACLF compared to NLF patients is plausible. As ascites was present in most ACLF patients, we assume that the ascites is the most important factor in the increase in *V*_2_. All patients in the NLF group as controls were admitted for sepsis or septic shock and it is known that *V*_2_ is already increased in these conditions [32]. Therefore, in ACLF patients *V*_2_ was even increased further above the increase in sepsis or septic shock.

No difference in the clearance of meropenem was found between the two groups. It has been shown before that 80 to 90% of meropenem and its metabolites are renally eliminated [23], that the clearance mainly depends on renal clearance [33, 34], and that liver impairment does not influence the clearance [11]. In our study cohort with critically ill patients, no difference in clearance could be shown either, confirming the previous results. Notably, in our patient population, the clearance of approximately 5 L/h was lower than 8.3 to 9.3 L/h as previously reported [35, 36], but comparable to a clearance of 4.8 L/h as shown for anuric patients receiving renal replacement therapy [37]. Therefore, the meropenem elimination in our cohort most likely relied solely on the renal replacement therapy in both groups. All other parameter values were comparable to those previously reported for critically ill patients [36]. A multitude of clinical studies of meropenem PK in healthy volunteers and critically ill patients, based on extensive sampling data, described meropenem concentration–time profiles as bi-exponential owing to the vast distribution in blood and into various tissues [38–40]. Hence, in agreement with previous analyses of meropenem PK, in our study a two-compartment model with linear clearance from the central compartment was employed to describe meropenem PK.

We defined target attainment as reaching a minimum concentration four times above the respective ECOFF values for 100% of the time at steady-state (day 7) and of 95% on day 1 to account for meropenem concentrations below 4×ECOFF during the initial increase at the beginning of the first infusion without negatively affecting target attainment. Although lower target values of 50% or 100% of the time above a single MIC, or 40%, 50%, and 70% above the fourfold MIC have been proposed [26, 41], several studies and a recent guideline support our choice of 100% *f*T > _4×ECOFF_ [28]: an optimized outcome could be shown for meropenem concentrations continuously exceeding 4.3-fold MIC for other beta-lactam antibiotics [42] and in vitro studies with *Pseudomonas aeruginosa* even determined the maximum activity of ceftazidime at concentrations of the 6.6-fold MIC [43]. No additional killing was shown when increasing the concentrations from 5- to 20-fold MIC [44]. This strengthens the recommendation for continuous attainment of four to eightfold MIC for adequate PTA [28].

Antibiotic concentrations are usually measured in serum and do not reflect the concentrations at the infectious focus that should be above the MIC to allow for a continuous bactericidal effect. Meropenem’s penetration for peritoneal fluid is of approximately 70% [31], but only 25% for lung epithelial lining fluid and lung tissue [45, 46] and 20% for cerebrospinal fluid [47]. In cases of low penetration, it has been doubted that sufficient concentrations are attained for a sustained bactericidal effect [45]. This may explain why higher concentrations than the MIC are required for a continuous bactericidal effect and improved outcome.

**Fig. 3:**
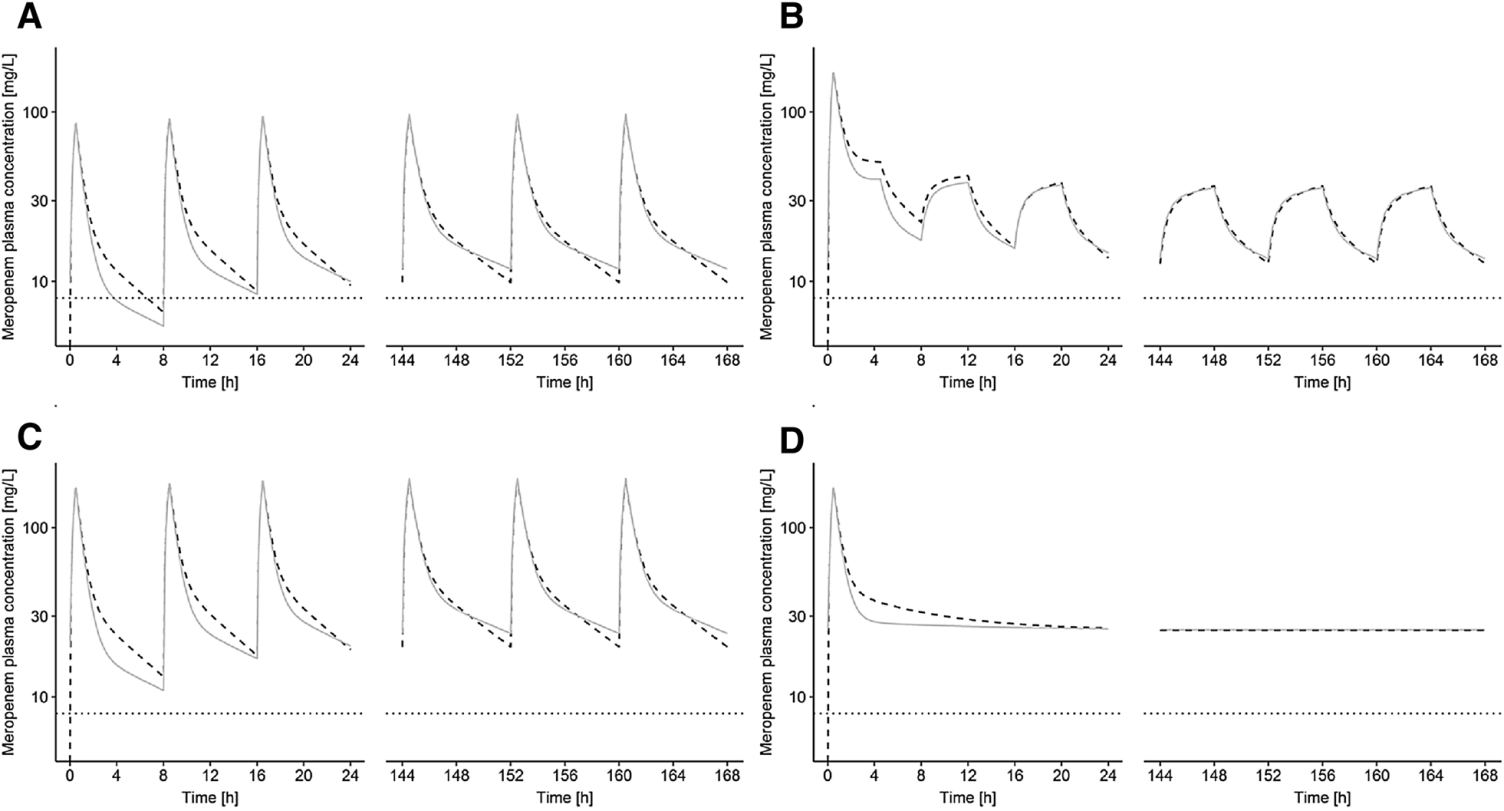
Deterministic simulations of meropenem. **a** 1 g quid 8 h (30 min); **b** 2 g loading dose (30 min) followed by 1 g prolonged infusion (over 4 h) quid 8 h; **c** 2 g quid 8 h (30 min); **d** 2 g loading dose (30 min) followed by continuous infusion of 3 g/day. Solid lines: typical patient without liver failure; dashed line: typical acute-on-chronic liver failure patient; dotted horizontal line depict typical target concentrations as 4x European Committee on Antimicrobial Susceptibility Testing epidemiological cut-off values (ECOFF) for *Pseudomonas* spp. (4× ECOFF 2.0 mg/L = 8 mg/L)

On the other hand, neurotoxicity is a known side effect of meropenem when high concentrations are maintained continuously. Steady-state minimum concentrations above 64 mg/L were shown to be associated with a 50% chance of developing neurotoxicity [48]. Our deterministic simulations suggested that our selected dosing regimens were safe as the trough concentrations were well below this threshold in the typical ACLF and NLF patient (Fig. 3).

It has been proposed that higher targets above the MIC also reduce the development of antibiotic resistance [49], but this concept has been challenged [50, 51] and further data are required.

With bolus application of beta-lactams, high peak concentrations are attained, but trough concentrations are often below the MIC or the targeted multiple of the MIC so that no continuous bactericidal effect is achieved. Patients’ outcome may improve with a longer time above the MIC [26, 52]. In patients with ACLF and a meropenem total dose of 3 g/day, PTA increased with a slower application of the respective 1 g aliquots, reaching 100% for the continuous infusion (Fig. 2).

Several attempts have been made to develop dosing nomograms and risk assessments for target non-attainment for meropenem in critically ill patients [33, 34, 36, 53, 54], but all approaches have mainly focused on the clearance at steady-state due to the primarily renal elimination. Although ACLF does not significantly alter clearance, the loading dose of meropenem needs special attention due to the increased V in ACLF patients. Our data strongly suggest administering a loading dose of 2 g meropenem in ACLF especially if *P. aeruginosa* or other non-fermenting bacteria with a high ECOFF or MIC are considered as causative organisms and to continue the therapy by continuous infusion to achieve concentrations above 4× ECOFF throughout the therapy. As soon as the steady-state is reached, no further adjustment of the meropenem dose is necessary in ACLF patients, as the PK parameters differ only insignificantly between the groups.

Our study has certain limitations. First, the number of patients was rather small and this necessarily limits the precision of the PK parameters. However, this is a common number of patients for pharmacokinetic studies and the first study assessing PK data in ACLF.

Second, we have decided to select high target concentrations with the use of the fourfold ECOFF as the basis for PTA calculations, but these concentrations are recommended by guidelines [28].

Third, we did not measure the free fraction of meropenem. However, meropenem is only negligible bound to albumin at 2% [38].

## Conclusion

We evaluated the PTA of meropenem in critically ill patients with ACLF undergoing CVVHD for the fourfold ECOFF values for *Enterobacterales* and *Pseudomonas* spp. For *Enterobacterales*, all simulated dosing regimens including the standard 1 g short-term infusion quid 8 h dosing regimen yielded a PTA of 100% and therefore resulted in adequate drug exposure. For *Pseudomonas* spp. only the dosing regimens of 2 g short-term infusion quid 8 h and the continuous dosing regimen of 3 g/day showed adequate PTA at steady-state. Compared to NLF patients who were suffering from sepsis or septic shock with an already increased *V*_2_ as compared to non-critically ill patients, ACLF patients showed an even higher *V*_2_ highlighting the importance of sufficient loading doses to attain adequate PTA on the first day of therapy. No dose adjustments in ACLF patients were necessary at steady-state. However, over time the course of antibiotic therapy, therapeutic drug monitoring of meropenem might aid in the guidance of optimal antibiotic treatment.

## Data Availability

Data are available from the authors upon reasonable request.

## Abbreviations

ACLF: Acute-on-chronic liver failure
CLIF: Chronic Liver Failure Consortium
CVVHD: Continuous veno-venous hemodialysis
ECOFF: European Committee on Antimicrobial Susceptibility Testing epidemiological cut-off values
EUCAST: European Committee on Antimicrobial Susceptibility Testing
*f*T > _4×ECOFF_: Time of the free antibiotic fraction above the fourfold of the European Committee on Antimicrobial Susceptibility Testing epidemiological cut-off value
MIC: Minimal inhibitory concentration
MELD: Model for end-stage liver disease
NLF: No liver failure
n.s.: Not statistically significant
PK: Pharmacokinetics
PTA: Probability of target attainment
SOFA: Sequential Organ Failure Assessment
*V*: Volume of distribution
*V*_1_: Central volume of distribution
*V*_2_: Peripheral volume of distribution
